# Psychosocial factors associated with malaria care-seeking in rural Ethiopia

**DOI:** 10.1186/s12889-022-13862-x

**Published:** 2022-08-01

**Authors:** Bolanle Olapeju, Habtamu Tamene, Minyahil Ayele, Simon Heliso, Tsega Berhanu, Guda Alemayehu, Nandita Kapadia-Kundu

**Affiliations:** 1 Department of Health, Behavior and Society, Johns Hopkins Bloomberg School of Public Health, Baltimore, USA; 2 Johns Hopkins Center for Communication Programs, Baltimore, USA; 3 Johns Hopkins Center for Communication Programs - Ethiopia, Addis Ababa, Ethiopia; 4 U.S. President’s Malaria Initiative, USAID, Addis Ababa, Ethiopia

**Keywords:** Care-seeking, Ethiopia, Malaria, Psychosocial

## Abstract

**Background:**

Ethiopia’s National Malaria Control and Elimination Program aims to diagnose all suspected malaria cases within 24 h of fever onset and provide prompt treatment for confirmed cases. This study explored psychosocial factors associated with no-, delayed- and prompt- care-seeking among female caregivers of children under five years with fever in rural Ethiopia.

**Methods:**

Household surveys were conducted from 2016–2019 among female caregivers (*N* = 479) of children under five years old with fever in Oromia; Amhara; Southern Nations, Nationalities, and Peoples Region (SNNPR); and Tigray. Prompt and delayed care-seeking were defined as seeking treatment within ≤ 24 h or > 24 h of symptom onset respectively. Contextual factors explored included sociodemographic factors, household supply of bed nets, exposure to health messages, and household vulnerability (a measure of financial access to food, shelter, schooling, and medical treatment). Ideational factors included psychosocial factors related to care-seeking (knowledge, self-efficacy, response efficacy, attitudes, involvement in decision-making, and household social support).

**Results:**

The prevalence of fever among children under five years was 18% (ranging from 9% in Tigray to 34% in SNNPR. Overall, 45% of caregivers of children with fever sought care promptly, while 23% delayed care-seeking and 32% sought no care. Prompt care-seeking rates were higher among caregivers with positive attitudes toward prompt care-seeking (48%), involved in decision-making (48%) or perceived equitable gender norms in the community (65%). Caregivers with a high care-seeking ideation had increased odds of prompt care-seeking (aOR: 2.65; 95% CI: 1.74–4.02). Significant contextual factors included residence in the Oromia region (aOR: 2.99; 95% CI:1.40–6.41), caregivers age 35–49 years (aOR: 0.49; 95% CI: 0.26–0.95), residence in vulnerable households (aOR: 2.01; 95% CI: 1.28–3.18).

**Conclusions:**

Among this rural Ethiopian population, prompt care-seeking was low but positively influenced by both ideational and contextual psychosocial factors occurring at the caregiver level. Multi-sectoral interventions at the individual, community, and health facility levels are needed to improve prompt care-seeking. These include social behavior change interventions to improve ideation, complemented by health facility interventions to ensure provision of high-quality services and structural interventions to increase educational attainment in these rural settings.

**Supplementary Information:**

The online version contains supplementary material available at 10.1186/s12889-022-13862-x.

## Background

In 2019, Ethiopia achieved the Global Technical Strategy for Malaria 2020 target of a 40% reduction in malaria case incidence and reduction in mortality rate [1]. While Ethiopia is characterized by an overall low malaria prevalence rate, over half of Ethiopians remain vulnerable to malaria with 27 million people living in high-transmission zones (more than one case per 1,000 population) [2] and over 250,000 confirmed cases of malaria in 2019 [1].

Appropriate management of fever cases, insecticide-treated net (ITN) use, and intermittent presumptive treatment of malaria in pregnancy remain key to malaria control. The National Malaria Control and Elimination Program (NMCEP) has made great strides in reducing malaria burden and charting a path to elimination, including the roll-out of rapid diagnostic testing (RDT) and artemisinin-based combination therapy at the community level [2]. Prompt care-seeking, defined as seeking care within the same day or next day of fever onset [3], is a key behavior to ensuring proper case management of suspected and confirmed malaria cases [4].

NMCEP aims to ensure all suspected malaria cases are diagnosed using either RDT or microscopy within 24 h of fever onset and all households living in malaria-endemic areas have the knowledge, attitudes, and practices necessary for adopting appropriate health-seeking behavior for malaria prevention and control [2]. While the 2015 National Malaria Indicator Survey demonstrated that advice or treatment was sought for half of all children with fever, the number of children with fever for whom care was promptly sought is unknown [3]. Considering NMCEP’s priorities and the critical role of prompt care-seeking in ensuring proper diagnosis and treatment of malaria, relevant stakeholders need to understand the factors influencing care-seeking behaviors for children with fever, particularly at the caregiver level [2].

Of particular interest is the role of caregivers’ psychosocial factors, defined as social, cultural, environmental phenomena, and other influences that may affect their behavior [5]. Psychosocial factors play an indubitable role in health and behavior change [6–8]. Recent studies in Ethiopia have explored the psychosocial factors associated with prompt care-seeking [9–12]. A facility-based study of a predominantly adult patient population in the Amhara region revealed that about half of febrile patient participants sought treatment within 24 h of fever onset, and this was higher among patients who were knowledgeable about malaria prevention and transmission, close to a health center, or had a family size of less than five household members [12]. In Tigray, care-seeking was delayed among low-income patients and those without health insurance [10]. In Dera, northwest Ethiopia, income, community-based health insurance, previous history of malaria infection, decision-making and distance from the facility were determinants of delay in seeking treatment for malaria [11]. In addition, a community based study in Jimma found higher rates of prompt care-seeking among Muslim and uneducated respondents [9].

However, few studies have specifically explored factors influencing prompt care-seeking among caregivers of febrile children under five years of age. This warrants deeper research, as children under five years remain more vulnerable to malaria complications. Community studies demonstrated low levels of caretakers' understanding of malaria in specific localities. Specifically, in the Mandura district of West Ethiopia, a considerable number of caregivers first consulted traditional healers and tried home treatment and thus, sought treatment late [13]. Caregivers’ ages, malaria knowledge, attitudes, perceived susceptibility to malaria, and perceived barrier to seek treatment, and if caregivers lived in rural villages, were important factors in seeking health care [13].

This study seeks to explore the spectrum of prompt care-seeking behavior and associated psychosocial factors among female caregivers of children under five years with fever in rural Ethiopia: Oromia; Amhara; Southern Nations, Nationalities, and Peoples Region (SNNPR); and Tigray. The study objective is to identify the extent to which Ethiopian caregivers who seek care promptly, differ from those who delay seeking care, and from those who never seek care at all. Study findings can inform the design of evidence-based community engagement approaches that appropriately target caregivers in these regions and other similar settings.

**Fig. 1:**
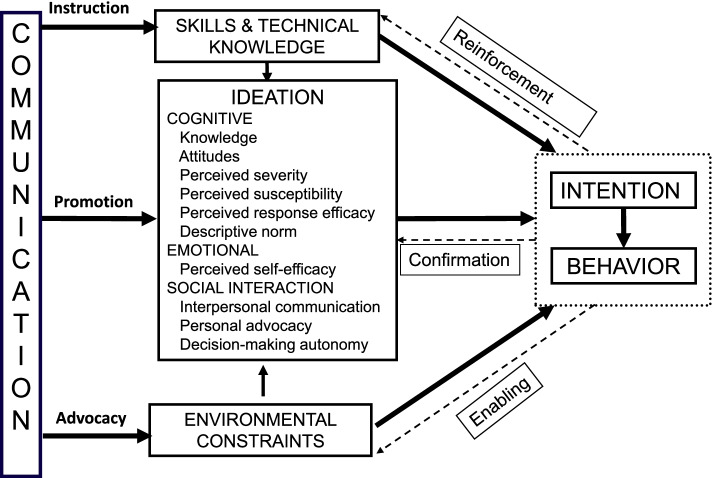
Ideation model of strategic communication and behavior change. Source: Adapted from Kincaid DL. Mass media, ideation, and behavior: A longitudinal analysis of contraceptive change in the Philippines. Comm Res. 2000;27(6):723–63

Several conceptual frameworks have explored psychosocial factors influencing health behavior. As shown in Fig. 1, the ideation framework developed by Kincaid serves as the underpinning for this manuscript [14, 15]. The concept of ideation has proven useful in understanding individual-level psychosocial factors affecting malaria-specific outcomes including ITN use [16, 17] and appropriate care-seeking [18]. The ideation framework is drawn from various behavioral theories and recognizes that most behavioral decisions are driven by psychosocial factors including cognitive, emotional, and social factors [15]. Cognitive factors include knowledge of disease symptoms; transmission and prevention; and attitudes, values, and attitudes related to proposed actions. Emotional factors include perceived severity and susceptibility to disease, perceived self-efficacy and belief in the efficacy of proposed actions. Social factors include social support, social influence, spousal/partner communication, and personal advocacy.

This study uses the ideation framework to understand the contextual and ideational psychosocial factors associated with care-seeking for malaria among caregivers of febrile children under five years in Ethiopia The study explores a limited number of ideational psychosocial factors related to care-seeking (malaria-related knowledge, self-efficacy, response-efficacy, attitudes, decision-making agency, community norms and social support in the household) against the backdrop of overarching contextual psychosocial factors (demographics, physical environment, and access to resources) seen among caregivers in Ethiopia.

## Methodology

### Study background

The data presented in this manuscript are drawn from survey data for the Communication for Health (C4H) project- a five-year (2015–2020) project in Ethiopia funded by the United States Agency for International Development focusing on integrated social and behavior change communication (SBCC) in multiple health areas including malaria [16]. The goal of the C4H project was to improve health practices through standardized SBCC interventions in four major regions of Ethiopia: Amhara, Oromia, Tigray, and SNNPR. The project conducted formative studies, including a baseline survey in 2016 targeting all woredas in the four study regions and a follow-up survey in 2019 assessing the exposure and effectiveness of the interventions among 160 woredas where the interventions had been implemented. The C4H project study population was women of reproductive ages (15–49 years) who were the primary beneficiaries of the project’s social and behavior change interventions. The C4H project implemented integrated social and behavior change activities including mass-media and interpersonal communication interventions targeting the general population. Health messages promoting timely care-seeking were included in radio spots, a weekly drama/education radio series; vans mounted with loudspeakers disseminate messages in intervention areas; offline videos focusing on postnatal care, essential newborn care, seeking care for newborn illnesses, immunization, and malaria prevention and treatment; a mobile application that contain key health messages including prevention and care seeking and complementary training of community health workers to conduct effective house to house education and outreach activities such as cue-cards and family health guide.

### Sampling strategy

The study data are drawn from two cross sectional studies in 2016 and 2019. In each round, C4H applied probability proportionate to size (PPS) sampling to select project woredas and enumeration areas (EAs). In each EAs, all households were listed to identify eligible households (with women aged 15–49) from which 35 eligible households were randomly selected. The C4H data collectors interviewed all women aged 15–49 face to face from the eligible households using electronic data collection tools. A total of 2,770 women were interviewed at baseline and 1,773 at the follow-up survey, because the intervention was implemented in fewer communities than the potential number determined at baseline. Among all women surveyed, 2453 were caregivers of children under five years out of which 479 caregivers had a child or children with a fever in the two weeks prior to the survey. See the study flow chart in Supplemental Fig. 1 for more details.

### Power calculation

Assuming a 5% type I error, the sample of 479 women caregivers of children under five years old who reported fever results in 86% power to estimate the percent of caregivers who sought care promptly with 7% precision (estimate ranging from 44 to 51%).

### Ethical considerations

The study received ethical approval from the Ethiopian Public Health Institute (EPHI) ethical review committee, Addis Ababa, Ethiopia, and the institutional review board (IRB) of the Johns Hopkins Bloomberg School of Public Health (JHSPH), Baltimore, Maryland, USA (JHSPH IRB # 00,007,138 and EPHI-IRB-173–2019). All study procedures were performed in accordance with the Declaration of Helsinki. Informed consent was obtained from all study participants. Participants less than 18 years provided informed assent after parental consent was obtained.

### Data collection

The study interviewed a total of 4,543 women aged 15–49 (94% response rate). Of these, 2,770 (92% response rate) were interviewed at baseline; because the intervention was implemented in fewer communities than the potential number determined at baseline, 1,773 (97% response rate) were interviewed at follow-up surveys. The questionnaires aimed to collect information on common themes, including sociodemographic characteristics, malaria related behavior and psychosocial factors, gender equitable norms, and access to health education messages. The study administered questionnaires in the Amharic, Oromia, and Tigrigna languages.

### Data analysis

The study measured participants’ care-seeking behaviors and psychosocial factors accounting for contextual factors, including demographic characteristics, vulnerability, and gender equitable norms. The survey asked all caregivers, “Has [child’s name] been ill with a fever at any time in the last two weeks?” Any care-seeking was defined using the question, “Did you seek advice or treatment for the fever from any source?” This study defined overall care-seeking as “prompt,” “delayed,” or “none,” based on the time elapsed before seeking care.

The key outcome variable was prompt care-seeking for fever; explored by the survey question, “How long after the onset of fever did you seek treatment?” This study defined prompt care-seeking as seeking treatment within 24 h or less and Delayed care-seeking as seeking treatment more than 24 h after the onset of fever.

Psychosocial factors explored in this study included ideational factors relevant to care-seeking as well as overarching contextual factors. Ideational factors include cognitive, social and emotional constructs, which are quantitatively assessed using a battery of Likert scale questions to capture these latent variables [19]. A limited number of ideational psychosocial factors related to care-seeking (knowledge, self-efficacy, response efficacy, attitudes, involvement in decision-making, and social support in the household) were explored as follows:

- Knowledge: The study assessed this by exploring participants’ awareness of malaria, signs and symptoms, cause, and prevention measures. Specific questions included the following:
  - ◦ “Have you ever heard of an illness called malaria?”
  - ◦ “What signs or symptoms would lead you to think a person has malaria?”
  - ◦ “What do you think is the cause of malaria?”
  - ◦ “How can someone protect themselves against malaria?”
- Self-efficacy: This refers to an individual's belief in one’s capacity to execute behaviors necessary to produce specific performance attainments [18]. This study assessed participants' self-efficacy for care-seeking and malaria prevention using the following statement: “I can take my child to treatment within 24 h of onset fevers, and I am able to have children under five years sleep under an ITN each night.”
- Response efficacy: This refers to a person's attitudes as to whether the recommended action step will avoid the threat. The study sought to understand if participants understood the benefits gained if they engaged in prompt care-seeking or malaria prevention behavior. This study proposed statements to participants like, “Having my children sleep under an ITN each night will prevent malaria,” and, “Seeking treatment for my under five children within 24 h of onset fever improves chances of recovery and survival.”
- Attitudes towards care-seeking: This study assessed this using the statement, “I should seek treatment for children under five years within 24 h of onset of fever.” Responses for self-efficacy, response efficacy, and attitudinal questions were recorded on a four-point Likert scale of “Strongly agree,” “Agree,” “Disagree,” and “Strongly disagree.”
- Involvement in decision-making: this study explored this using the question, “Who usually makes decisions about health care for yourself: you, your (husband/partner), or you and your husband/partner jointly?” Respondents who noted they made decisions by themselves or jointly with their partners were considered involved in decision making.
- Social support: This study assessed this by asking about the level of spousal support on household chores using the question, “Does your husband help you with household chores like looking after the children, cooking, cleaning the house, and doing other work around the house?” This question served as a proxy for actual spousal support on caregiving.
- Community norms: This study measured caregivers’ perceptions regarding gender equitable norms using a 21-item Gender Equitable Men scale validated in Ethiopia [20]. The scale measure attitudes toward gender norms in intimate relationships or differing social expectations for men and women. It includes four sub scales related to physical violence, sexual relationships, reproductive health and disease prevention, and domestic chores and daily life. Responses to all 21 questions were condensed into a composite score, split into tertiles, and then subdivided into two categories of perceived gender equity: high perceptions of equitable gender norms (first tertiles) and moderate or low perceptions of equitable gender norms (second and third tertile).

This study condensed all ideational psychosocial variables into a composite score, referred to as care-seeking ideation, ranging from 0–12 (Cronbach's alpha of 0.7). Caregivers with a care-seeking ideation score of 9 or more (greater than the median) were categorized as having a high care-seeking ideation, while those with a score of 8 or less were categorized as having low care-seeking ideation. Supplemental Table 1 summarizes the psychosocial variables used in generating the malaria care-seeking ideation.

The study also explored the following overarching contextual psychosocial variables: caregivers’ region (Amhara, Oromia, SNNPR, Tigray), residence (urban versus rural), malaria transmission zone (high versus low/no), age (15–24, 25–34, and 35–49 years old), education (none versus primary or higher), religion (Christian versus non-Christian), marital status (married or cohabiting versus not), and wealth quintile based on ownership of household assets (richest, richer, middle, poorer, poorest).

Access to ITNs within the household was defined as the number of bed nets per household member. Given the assumption that a bed net can be used by up to two people [21], a value of ≥ 0.5 bed nets per household member was defined as adequate ITN supply, while < 0.5 was classified as inadequate. Exposure to health communication messages was assessed by asking whether caregivers had the *Family Health Guide* (FHG), a national health communication tool given to all families. FHG comprises more than 79 messages with cues to action and illustrations focusing on multiple health areas including malaria.

Additionally, a vulnerability index was assessed in the survey using four states the participant said they experienced in the last 12 months: (1) lacked adequate nutrition, (2) lacked shelter/house to stay in, (3) was unable to afford to send children to school, and (4) lacked sufficient money to buy medicines/medical treatment. Responses included never (coded as 1), rarely (coded as 2), sometimes (coded as 3), and often (coded as 4). We computed a composite score from the sum of the four responses. The vulnerability index was divided into two categories: non-vulnerable (score ≤ 7) and vulnerable (8–16), with high scores indicating a greater level of poverty. Finally, the study included cross-sectional survey timing (2016 baseline versus 2019 follow-up) as a contextual variable to account for temporal changes that might explain inter relationships between psychosocial factors and care-seeking.

Since we performed analysis on children under five years with a fever in the two weeks preceding the survey, we explored any clustering effect that might occur with multiple children under five years in the same household/caregiver. No evidence of clustering in the data (intraclass correlation = 0.015) appeared. The study used chi-square, t-tests, Analyses of Variance for bivariable tests of association and exploratory analysis, to compare psychosocial factors across the spectrum of care-seeking. We employed multivariable logistic regressions to explore psychosocial factors associated with both care-seeking and prompt care-seeking. Data management and analysis was performed using Stata software, version 16 (Stata Corporation, College Station, TX, USA) and Excel 2016 (Microsoft Corp, Seattle, WA, USA). We weighted the data using the svyset command in Stata created from the probabilities of woreda, EA and household selection to ensure the data was representative of the study population.

## Results

### Spectrum of care-seeking for children with fever

Table 1 highlights the spectrum of care-seeking for children with fever and caregiver’s contextual psychosocial factors. On average, caregivers were aged 25–34 years old (48%, 229/479), identified as Christian (64%, 305/479), and were married (94%, 452/479). While only few caregivers resided in households considered to be vulnerable (27%, 131/479), minimal households had adequate mosquito nets (27%, 131/479) or had the FHG (11, 55/479%).

**Table 1:** Spectrum of care-seeking and contextual psychosocial factors (*N* = 479)

Characteristics	Total		No care		Delayed care		Prompt care		*P* value
	No	%	No	%	No	%	No	%	
**Total**	**479**	**100**	**153**	**32**	**112**	**23**	**214**	**45**	**n/a**
**Region**									**0.001**
Amhara	131	27	60	40	28	25	43	20	
Oromia	146	31	31	20	37	33	79	37	
SNNPR	160	34	43	28	43	38	75	35	
Tigray	41	9	19	12	5	4	17	8	
**Malaria transmission**									0.648
Free/Low	166	35	55	36	34	30	78	36	
Moderate/High	313	65	98	64	78	70	137	64	
**Age in years**									0.189
15–24	82	17	23	15	15	14	44	20	
25–34	229	48	71	47	48	43	109	51	
35–49	168	35	59	38	48	43	62	29	
** ≥ Primary education**	154	32	37	24	28	25	89	41	0.005
**Christian religion**	305	64	105	69	75	67	125	58	0.168
**Married**	452	94	143	94	109	97	200	93	0.432
**Wealth Index**									0.413
Poorest	128	27	53	34	24	22	51	24	
Poorer	53	11	20	13	14	12	20	9	
Middle	114	24	36	23	26	23	53	25	
Richer	73	15	20	13	17	15	37	17	
Richest	111	23	25	16	31	28	54	25	
**Household has adequate nets**	63	13	15	10	14	12	34	16	0.273
**Vulnerable household**	131	27	44	29	21	18	67	31	0.103
**Household owns family health guide**	55	11	18	12	8	7	29	14	0.235
**Survey timing**									0.600
Baseline (2016)	311	65	103	67	67	60	140	65	
Follow up (2019)	168	35	50	33	44	40	74	35	

Overall, 45% of caregivers of children with fever sought care promptly while 23% delayed care-seeking and 32% sought no care. Care-seeking behavior differed significantly by some contextual psychosocial factors. Prompt care-seekers were more observed in Oromia (37%, 79/214) and SNNPR (35%, 75/214), compared to Amhara (20%) and Tigray (8%) regions. Caregivers’ education significantly influenced care-seeking behavior as 41% (89/214 of prompt care-seekers had formal education (only about a quarter of women who delayed or sought no care had a formal education). There were no significant differences in the spectrum of care-seeking by malaria transmission, age, religion, marital status, wealth index, household net supply, vulnerability status, ownership of the health guide or survey timing.

### Ideational psychosocial factors related to care-seeking

Ideational psychosocial factors explored included knowledge, self-efficacy, response efficacy, attitudes, decision making involvement and social support in the household. Table 2 shows the spectrum of care-seeking and associated ideational psychosocial factors. While the majority (95%) of caregivers were aware of malaria, only 52% knew malaria symptoms, 60% knew the cause of malaria and 24% knew measures to prevent malaria. Most caregivers had positive attitudes towards prompt care-seeking (90%). In addition, most caregivers had positive perceptions related to malaria including perceived response efficacy for prompt care-seeking (96%) and malaria prevention (88%), perceived self-efficacy to seek care promptly (87%) and prevent malaria (77%). Many (70%) caregivers reported involvement in decision-making, but few noted social support in the household from their partners (44%). Overall, less than half (44%) of caregivers possessed high care-seeking ideation.

**Table 2:** Spectrum of care-seeking and ideational psychosocial factors (*N* = 479)

Care-seeking ideational factors	Total (*N* = 479)		No care (*N* = 153)		Delayed care (*N* = 112)		Prompt care (*n* = 214)		*P* value
	**No**	**%**	**No**	**%**	**No**	**%**	**No**	**%**	
**Ever heard of malaria**	453	95	145	95	106	95	202	94	0.952
**Knowledge of malaria symptoms**	250	52	76	49	56	50	118	55	0.637
**Knowledge of malaria cause**	286	60	85	56	63	56	138	64	0.327
**Knowledge of malaria prevention measures**	115	24	35	23	24	22	56	26	0.745
**Perceived self-efficacy for prompt care-seeking**	415	87	120	79	90	80	205	96	** < 0.001**
**Perceived self-efficacy for malaria prevention**	370	77	112	73	88	78	170	79	0.527
**Perceived response efficacy of prompt care seeking**	462	96	146	96	106	95	209	98	0.514
**Perceived response efficacy of malaria prevention**	423	88	124	82	102	91	196	92	**0.020**
**Positive attitudes toward prompt care-seeking**	432	90	128	84	97	87	208	97	**0.001**
**Involvement in decision making**	333	70	110	72	63	56	160	74	**0.016**
**Social support in household**	211	44	69	45	46	41	97	45	0.797
**Perceived equitable gender norms**	68	14	12	8	12	11	44	20	**0.010**
**Overall high care-seeking Ideation**	210	44	54	35	39	35	117	55	**0.002**

Specific ideational psychosocial factors significantly associated with care-seeking included self-efficacy for prompt care-seeking, response efficacy of malaria prevention, positive attitudes toward prompt care-seeking, involvement in decision-making, and perceived equitable gender norms in the community. Specifically, the overwhelming majority of caregivers who sought care promptly had perceived self-efficacy for prompt care-seeking (96%, 205/214) compared to their counterparts who delayed (80%,90/112) or did not seek any care (79%, 120/153). Similarly, caregivers who sought care promptly were more likely to have perceived response efficacy of malaria prevention (92%, 196/214) compared to those who did not seek care (82%, 124/153). The majority of caregivers who sought care promptly for their children with fever had positive attitudes towards care-seeking (97%, 208/214)) compared to those delayed (87%) or no (84%) care-seeking. Caregivers who sought care promptly were more likely to be involved in household decision-making (74%, 160/214) or perceived equitable gender norms in their community (20%, 44/214) compared to caregivers who delayed care-seeking or sought no care.

Overall, the study observed significantly higher rates of overall high care-seeking ideation among caregivers with prompt care-seeking (55%, 117/214) compared to caregivers with delayed or no care-seeking (35% respectively).

### Contextual psychosocial factors associated with high prompt care-seeking ideation

Table 3 highlights the contextual psychosocial factors associated with high prompt care-seeking ideation. These included malaria transmission zone, age, and marital status. Specifically, while caregivers in SNNPR had reduced odds of a high care-seeking ideation (adjusted odds ratio (aOR): 0.39; 95% confidence interval (CI): 0.19–0.77), caregivers in high transmission zones had increased odds of having high levels of care-seeking ideation (aOR: 1.85; 95% CI: 1.03–3.30). Older caregivers aged 25–34 years (aOR: 2.41; 95% CI: 1.32–4.38) and 35–49 years (aOR: 2.04 (1.05–3.93) old had increased odds of a high care-seeking ideation compared to caregivers aged 15–24 years. In addition, caregivers with a primary education (aOR: 2.24; 95% CI: 1.42–3.55) had increased odds of a high care-seeking ideation.

**Table 3:** Contextual psychosocial factors associated with high prompt care-seeking ideation (*N* = 479)

Contextual psychosocial factors	High care-seeking ideation (Prevalence = 44%, 210/479)			
	OR	95% CI	aOR	95% CI
**Region**				
Amhara (reference)	1.00	N/A	1.00	N/A
Oromia	0.40	0.22–0.72	0.54	0.25–1.13
SNNPR	0.72	0.42–1.22	**0.39**	**0.19–0.77**
Tigray	1.24	0.73–2.09	0.95	0.52–1.75
**High Malaria transmission zone** (reference- no/low)	**1.98**	**1.31–2.99**	**1.85**	**1.03–3.30**
**Age in years**				
15–24 (reference)	1.00	N/A	1.00	N/A
25–34	1.58	0.94–2.66	**2.41**	**1.32–4.38**
35–49	1.29	0.74–2.22	**2.04**	**1.05–3.93**
**Primary education or more** (reference- none)	**1.99**	**1.36—2.92**	**2.24**	**1.42–3.55**
**Non-Christian** (reference-Christian)	**0.47**	**0.32–0.71**	0.79	0.45–1.37
**Married** (reference- Not married)	1.56	0.73–3.34	1.54	0.68–3.49
**Wealth index**				
Poorest (reference)	1.00	N/A	1.00	N/A
Poorer	0.68	0.36–1.29	0.78	0.39–1.54
Middle	0.81	0.49–1.34	1.00	0.57–1.76
Richer	1.16	0.66–2.03	1.42	0.74–2.72
Richest	1.01	0.61–1.69	1.44	0.74–2.81
**Household has adequate bed nets** (reference- no)	1.46	0.88–2.42	1.05	0.60–1.83
**Vulnerable household** (reference- no)	0.73	0.48–1.09	0.86	0.55–1.35
**Household owns family health guide** (reference-no)	1.04	0.64–1.68	0.75	0.44–1.27
**Survey timing**				
Baseline (2016)	1.00	N/A	1.00	N/A
Follow up (2019)	1.06	0.73–1.54	0.86	0.56–1.31

Table 4 details the inter-relationship between prompt care-seeking behavior and contextual psychosocial factors. Caregivers with a high care-seeking ideation had increased odds of prompt care-seeking (aOR: 2.65; 95% CI: 1.74–4.02) compared to their counterparts with low care-seeking ideation. Contextual psychosocial factors associated with prompt care-seeking include residence in Oromia region (aOR: 2.99; 95% CI: 1.40–6.41), being educated (aOR: 1.71; 95% CI: 1.07–2.72), and residence in a vulnerable household (aOR: 2.01; 95% CI: 1.28–3.18). On the other hand, older caregivers aged 35–49 years old had lower odds of prompt care-seeking (aOR: 0.49; 95% CI: 0.26–0.95).

**Table 4:** Inter-relationship between prompt care-seeking behavior, ideational and contextual psychosocial factors (*N* = 479)

	**Prompt care-seeking** **(Prevalence = 45%)**			
	OR	95% CI	aOR	95% CI
**Ideational psychosocial factors**				
High care-seeking ideation (reference-low)	**2.12**	**1.46–3.06**	**2.65**	**1.74–4.02**
**Contextual psychosocial factors**				
**Region**				
Amhara (reference)	1.00	N/A	1.00	N/A
Oromia	**2.19**	**1.23–3.91**	**2.99**	**1.40–6.41**
SNNPR	**1.79**	**1.04–3.10**	1.77	0.86–3.65
Tigray	1.27	0.73–2.20	1.17	0.61–2.25
**High Malaria transmission zone** (reference- no/low)	0.84	0.57–1.26	0.85	0.46–1.55
**Age in years**				
15–24 (reference)	1.00	N/A	1.00	N/A
25–34	0.79	0.48–1.32	0.64	0.36–1.15
35–49	**0.53**	**0.31–0.91**	**0.49**	**0.26—0.95**
**Primary education or more** (reference- none)	**1.87**	**1.27–2.73**	**1.71**	**1.07–2.72**
**Non-Christian** (reference-Christian)	1.34	0.90–1.97	0.93	0.53–1.65
**Married** (reference- Not married)	0.67	0.32–1.39	0.60	0.27–1.33
**Wealth index**				
Poorest (reference)	1.00	N/A	1.00	N/A
Poorer	1.23	0.65–2.33	1.29	0.65–2.57
Middle	1.58	0.95–2.61	1.41	0.79–2.51
Richer	**2.04**	**1.15–3.60**	**2.08**	**1.07–4.02**
Richest	1.55	0.92–2.61	1.29	0.65–2.54
**Household has adequate bed nets** (reference- no)	1.30	0.78–2.15	1.43	0.80–2.54
**Vulnerable household** (reference- no)	**1.50**	**1.00–2.25**	**2.01**	**1.28–3.18**
**Household owns family health guide** (reference-no)	1.07	0.66–1.74	1.47	0.85–2.53
**Survey timing**				
Baseline (2016)	1.00	N/A	1.00	N/A
Follow up (2019)	0.79	0.54–1.16	0.75	0.49–1.15

## Discussion

### Principal findings

This study is of significance as it is one of few studies exploring factors associated with prompt care-seeking for children under five years with fever in rural Ethiopia. Among the rural Ethiopian population, prompt care-seeking rates were low but positively associated with both ideational and contextual psychosocial factors occurring at the caregiver level. The study found an association between high care-seeking ideation and higher rates of prompt care-seeking. Specific ideational factors of note in this setting included perceived self-efficacy, response efficacy, attitudes toward prompt care-seeking, and involvement in household decision-making. Contextual psychosocial factors associated with influencing prompt care-seeking in rural Ethiopia included region of residence, education, and household vulnerability. This study reported similar rates of care-seeking ideation and behavior across the baseline and follow-up surveys. Of note, a C4H program evaluation report demonstrated significantly higher rates of prompt care-seeking, knowledge, and perceived efficacy among caregivers exposed to the program interventions [22].

The findings are consistent with similar studies in Ethiopia. With regards to ideational factors such as knowledge, perceived self-efficacy and response efficacy, studies in Southwest Ethiopia demonstrated that mothers who have poor knowledge [23], negative attitudes about malaria treatment [24] or misperceptions about the effectiveness of antimalarial drugs [9] were more likely to delay treatment for children under five with fever. Furthermore, in Northwest Ethiopia, decisions to seek health care were found to be taken mostly by fathers, which in turn resulted in delayed care seeking [10]. Study findings on contextual factors such as region, education and household status influencing prompt care seeking in this study were corroborated with studies in West and Northwest Ethiopia affirming that place of residence [13, 25], and education [10] were key determinants of health seeking. In other settings across sub-Saharan Africa, women’s education [26], household income [27], and reduced perceived efficacy of treatment [27] were significant correlates of care seeking. To the best of our knowledge, this is the first study exploring the interconnections between care-seeking and both ideational psychosocial or contextual factors in Ethiopia, employing a larger perspective and elucidating the complementarity of both psychosocial and structural factors associated with malaria related behavior.

### Study implications

Study findings can inform relevant behavior change, structural and research interventions to reverse this trend in rural Ethiopia. This study demonstrated the overall role of psychosocial ideational factors. A key premise behind the ideation model is that people do not ordinarily take action, especially new action, until they have gained sufficient knowledge about it and its consequences, until they have a positive attitude toward it, and until they have talked to others about it and feel right about it [14].

Communication can directly influence such cognitive, emotional, and social factors of ideation separately, as well as jointly as these ideational factors often are interdependent and often occur simultaneously. Considering this, social and behavior change interventions should employ messaging that not only improves communities’ knowledge about prompt care-seeking and its benefits but also promotes a positive attitude about prompt care-seeking, generates conversation and decision-making regarding prompt care-seeking, and enables communities to feel right about seeking care promptly. Behavior change approaches should aim to promote supportive norms within the community and remove barriers to caregivers’ self-efficacy and response efficacy regarding prompt care-seeking and other malaria related behavior. Such behavior change approaches to promote utilization of health services are more impactful when complemented with interventions to ensure high quality service delivery.

The role of contextual and ideational factors suggests interventions should employ a holistic approach to health and quality of life, not just focus on malaria. This is of critical importance, as educational achievement and gender equality norms remain key factors in rural Ethiopia. Holistic approaches should employ a multi-sectoral lens and combine health, education, and gender empowerment interventions to improve the lives of community members. Investing in women, and, more specifically, girl's education, has several positive effects on children, women, and communities at large, combating poverty and fostering economic growth [28, 29]. At the heart of achieving gender equality is the education of girls and women and the removal of barriers to education and opportunities for their advancement.

### Strengths and limitations

A key strength of the study is the use of a theory-driven, comprehensive exploration of psychosocial factors specific to a high-risk population. We expect that our study findings might be generalizable to similar rural contexts in sub-Saharan Africa. However, some study limitations include its cross-sectional design, which limits the ability to make causal inferences. The use of self-reported data from multiple survey rounds may be limited by recall or social desirability bias. While the study employed the ideation model as part of its theoretical underpinnings, some aspects of this theory such as social influence, spousal/partner communication, and personal advocacy were not explored in this study. In addition, the study did not ask about health provider- or facility-level factors that could influence care-seeking, such as either perceived quality of care or stock out of commodities. Future research should seek to use prospective studies to validate findings with more complete measures of the ideational constructs.

## Conclusion

This study identified psychosocial factors among female caregivers of children under five years with fever seeking care in Ethiopia. Structural factors included caregivers’ level of education while ideational factors included caregivers’ self-efficacy and response efficacy, attitudes related to prompt care-seeking, involvement in decision-making, and perceptions of gender equitable norms in the community. Such factors should be accounted for when designing and implementing multi-sectoral interventions at community level.

## Supplementary Information

**Additional file 1: Supplemental Figure 1.** Study Participants Flow Chart, Flow diagram of participants included in the study

**Additional file 2: Supplemental Table 1.** Ideational psychosocial variables related to care seeking.

## Data Availability

The datasets used and analyzed during the current study are available from the corresponding author on reasonable request.

## Abbreviations

aOR: Adjusted odds ratio
C4H: Communication for Health
CI: Confidence interval
EA: Enumeration area
FHG: Family Health Guide
ITN: Insecticide-treated net
NMCEP: Ethiopia’s National Malaria Control and Elimination Program
PPS: Probability proportionate to size
RDT: Rapid diagnostic testing
SBCC: Social and behavior change communication
SNNP: Southern Nations, Nationalities, and Peoples Region
USAID: United States Agency for International Development
