# Supplementary material for: Psychosocial factors associated with malaria care-seeking in rural Ethiopia
Source: BMC Public Health. 2022 Aug 1;22:1460. doi: 10.1186/s12889-022-13862-x (PMC9341112; doi:10.1186/s12889-022-13862-x)
Supplement: Supplementary file 1 — Additional file 1: Supplemental Figure 1. Study Participants Flow Chart, Flow diagram of participants included in the study [file 12889_2022_13862_MOESM1_ESM.pptx]

## Slide 1
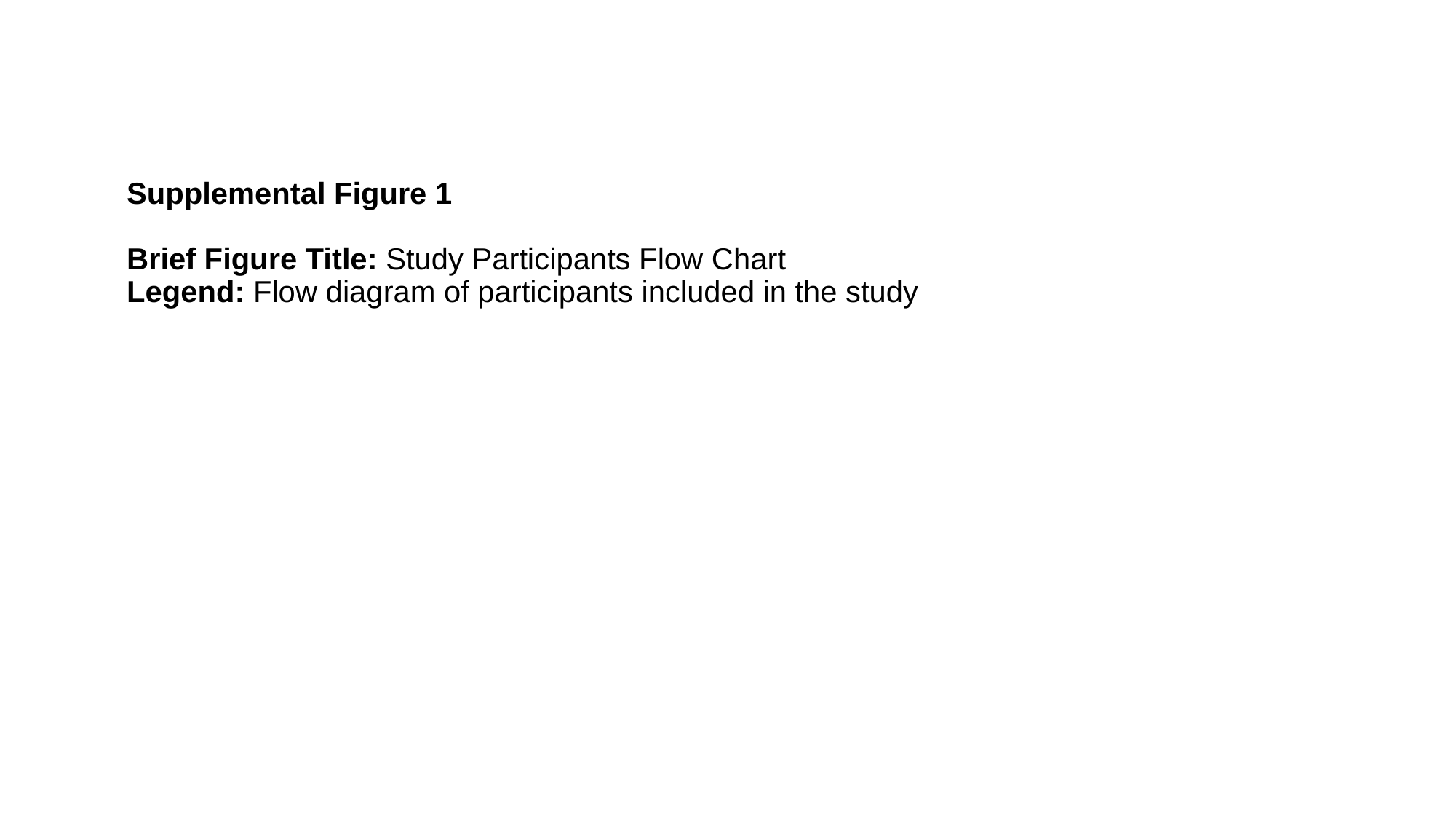

# Supplemental Figure 1 Brief Figure Title: Study Participants Flow Chart Legend: Flow diagram of participants included in the study

## Slide 2
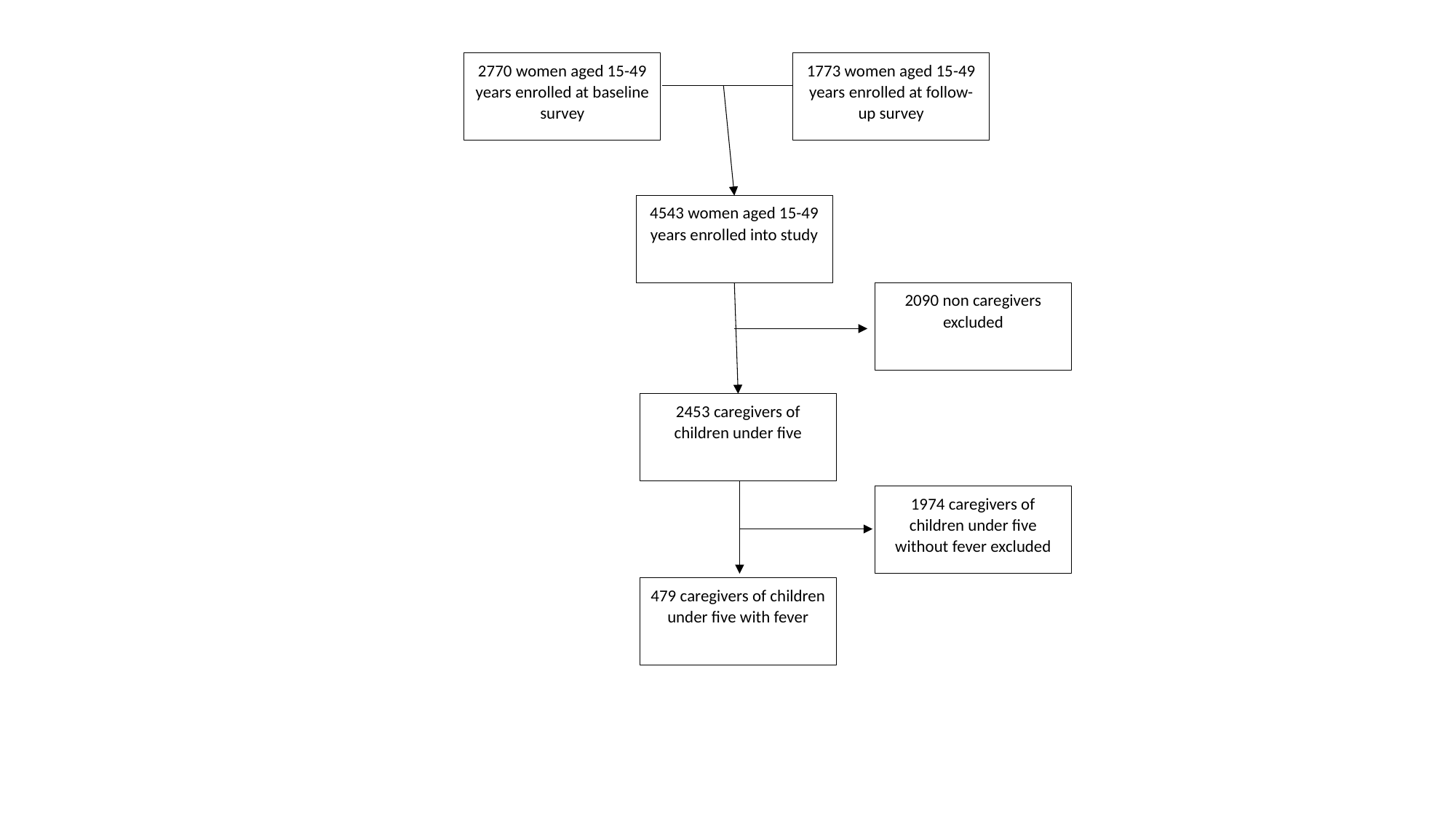

2770 women aged 15-49 years enrolled at baseline survey
1773 women aged 15-49 years enrolled at follow-up survey
4543 women aged 15-49 years enrolled into study
2453 caregivers of children under five
1974 caregivers of children under five without fever excluded
479 caregivers of children under five with fever
2090 non caregivers excluded
