# Supplementary material for: Psychosocial factors associated with malaria care-seeking in rural Ethiopia
Source: BMC Public Health. 2022 Aug 1;22:1460. doi: 10.1186/s12889-022-13862-x (PMC9341112; doi:10.1186/s12889-022-13862-x)
Supplement: Supplementary file 2 — Additional file 2: Supplemental Table 1. Ideational psychosocial variables related to care seeking. [file 12889_2022_13862_MOESM2_ESM.docx]

**Additional Files: Supplemental Table 1**

**Title: Ideational psychosocial variables related to care seeking**

| **Survey Questions** | **Responses** |
| --- | --- |
| **Awareness of malaria** |  |
| Have you ever heard of an illness called malaria? | Yes |
| **Knowledge of malaria symptoms** |  |
| What signs or symptoms would lead you to think a person has malaria? | Three or more correct symptoms^1^ |
| **Knowledge of the cause of malaria** |  |
| What do you think is the cause of malaria? | Mosquito bites |
| **Knowledge of malaria prevention measures** |  |
| How can someone protect themselves against malaria? | Three or more correct measures^2^ |
| **Perceived self-efficacy** |  |
| I am able to have children under five years sleep under an ITN each night. | (Strongly) agree |
| I can take my child to treatment within 24 hours of onset fever. | (Strongly) agree |
| **Perceived response-efficacy** |  |
| Having my children sleep under an ITN each night will prevent malaria for them. | (Strongly) agree |
| Seeking treatment for my children who are under five within 24 hours of onset fever improves chances of recovery and survival. | (Strongly) agree |
| **Beliefs related to prompt care-seeking** |  |
| I should seek treatment for children under five years within 24 hours of onset of fever. | (Strongly) agree |
| **Involvement in decision-making** |  |
| Who usually makes decisions about health care for yourself: you, your (husband/partner) or you and your husband/partner jointly? | Respondent or jointly with partner |
| **Social support in the household** |  |
| Does your husband help you with household chores like looking after the children, cooking, cleaning the house, and doing other work around the house? | Yes |
| **Community norms/ Perceived gender equitable norms** |  |
| Adapted Gender Equitable Men Scale with 21 statements on perceptions of physical violence, sexual relationships, reproductive health and disease prevention, and domestic chores and daily life. | Categorized as low versus moderate/high based on sum of correct responses. |
| ^1^ Symptoms include: Fever, Feeling cold, Headache, Nausea and vomiting, Diarrhea, Dizziness,  Loss of appetite, Body ache or joint pain, Pale eyes, Feeling weak, Refusing to drink.  ^2^ Measures include: sleep under a mosquito net, Sleep under an insecticide-treated mosquito net, Use mosquito repellant, Avoid mosquito bites, Take preventive medication, Spray house with insecticide, Use mosquito coils . | |
